# Mind the gap: Ongoing inequalities in glycaemic levels in young people living with Type 1 diabetes across England and Wales

**DOI:** 10.1111/dme.70283

**Published:** 2026-03-31

**Authors:** Victoria Homer, Cillian Brophy, Robert French, Amani Krayem, Saira Pons Perez, Justin T. Warner, Renuka P. Dias

**Affiliations:** ^1^ Cancer Research UK Clinical Trials Unit (CRCTU), Institute of Cancer and Genomic Sciences University of Birmingham Birmingham UK; ^2^ Birmingham NIHR Biomedical Research Centre Birmingham UK; ^3^ National Paediatric Diabetes Audit Royal College of Paediatrics and Child Health London UK; ^4^ School of Medicine Cardiff University Cardiff UK; ^5^ Noah's Ark Children's Hospital for Wales University Hospital of Wales Cardiff UK; ^6^ Department of Endocrinology and Diabetes, Birmingham Children's Hospital Birmingham Women's and Children's NHS Foundation Trust Birmingham UK; ^7^ Institute of Applied Health Research, College of Medical and Dental Sciences University of Birmingham Birmingham UK

**Keywords:** ethnicity, HbA1c, health inequalities, socio‐economic deprivation, type 1 diabetes

## Abstract

**Aims:**

Inequalities in glycaemic levels in children and young people (CYP) living with Type 1 diabetes (T1D) across different ethnic and socio‐economic groups in England and Wales were first highlighted in 2016. Almost 10 years on, we wanted to know if the gap has been closed.

**Methods:**

Analysis of 27,919 CYP with T1D from the 2022–2023 National Paediatric Diabetes Audit (NPDA). Multivariable linear regression was used to assess any association between socio‐economic status (SES), ethnicity or insulin pump use and HbA1c, adjusting for age, gender and diabetes duration.

**Results:**

CYP from ethnic minority groups continue to have significantly higher mean HbA1c levels compared to White CYP with the largest difference in Black children (6.8 mmol/mol, [0.6%], 95% CI 5.7, 7.9 mmol/mol). Lower SES remains associated with higher HbA1c levels with the largest difference between the least deprived and most deprived (7.0 mmol/mol; [0.6%], 95% CI: 6.4, 7.6). CYP from ethnic minority groups (Black 34.2% vs. White 49.56%, *p* < 0.0001) and those living in more deprived neighbourhoods (Least Deprived 54% vs. Most Deprived 41%, *p* = 0.0001) were less likely to use insulin pumps.

**Conclusions:**

Ethnicity and SES remained significantly associated with HbA1c levels even after accounting for the use of insulin pumps. Black CYP continue to have the highest glycaemic levels, and lower SES remains associated with poorer HbA1c outcomes. Substantial inequalities persist in the use of insulin pump therapy highlighting the ongoing need for targeted interventions to improve equity in diabetes care and outcomes.

## Graphical abstract

Inequalities in glycaemic outcomes for young people living with Type 1 Diabetes in England and Wales.

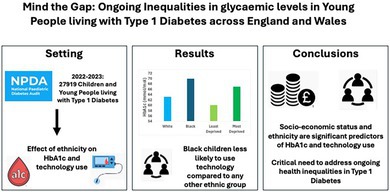

**What is already known?**

- 10 years ago, it was demonstrated that in England, glycaemic levels were higher in young people from ethnic minority groups and those living in the most deprived neighbourhoods.

**What has this study found?**

- Persistence of higher HbA1c in Black children when adjusted for socio‐economic status (SES) and technology use
- Lowest uptake of diabetes technology in Black children with no variation based on SES unlike other ethnic groups

**What are the implications of this study?**

- Children from underserved communities have higher HbA1c and are less likely to use technology compared to White peers; this needs to be addressed through appropriate and targeted awareness campaigns at a national and local level.

## 1 INTRODUCTION

Type 1 diabetes is the commonest cause of diabetes in children and young people, affecting approximately 1.5 million young people under the age of 20 years worldwide, and the incidence is rising, which poses a growing public health challenge.^1^, ^2^ However, children and young people from ethnic minority and/or more deprived communities have worse health outcomes. These include higher long‐term blood glucose level metrics (HbA1c), increased hospital admissions for diabetic emergencies (diabetic keto‐acidosis—DKA) and reduced use of diabetes technology. These disparities are not only evident in the United Kingdom but also seen internationally.^3^, ^4^, ^5^, ^6^ ^,^ ^7^ Poor glycaemic management tracks from adolescence into adulthood with long‐term impacts including reduced life expectancy and vascular complications in adulthood compared to peers without diabetes.^8^, ^9^, ^10^

In England and Wales, almost 35,000 children and young people under the age of 19 years live with Type 1 Diabetes.^11^ Over the past 10 years, there has been significant improvement in glycaemic levels across this cohort, with median HbA1c decreasing from 71.6 mmol/mol (8.7%, 2012–2013) to 60.5 mmol/mol (7.7%, 2022–2023).^11^ In that same time, children within the most deprived quintile have shown a 10.1 mmol/mol (0.9%) reduction in median HbA1c compared to children within the least deprived quintile who have shown a 10.0 mmol/mol (0.9%) reduction in median HbA1c over the same time (2012–2013 vs. 2022–2023 audit period).^11^ Similarly, across ethnic groups, there has been improvements in HbA1c with a reduction in mean HbA1c of 9.9 mmol/mol (0.9%) in White children vs. 11.1 mmol/mol (1%) in Black children (2012–2013 vs. 2022–2023 data).^11^

Despite these overall improvements across all young people living with Type 1 Diabetes, the large relative gap in glycaemic levels between groups persists.^11^ The difference in HbA1c between the most deprived and least deprived groups remains relatively static (11.5% 2022–2022 vs. 10% 2012–2013), and White vs. Black (10.7% in 2022–2023 vs. 10.2%). Similar inequalities have been previously reported.^12^, ^13^

Use of technology has been shown to positively impact on HbA1c outcomes and has also been shown to improve quality of life measures in young people.^14^ Diabetes technologies for people living with Type 1 diabetes are funded as per the National Institute for Clinical Excellence (NICE) guideline (NG18) and this was updated in 2022 (to offer real‐time or flash continuous glucose monitoring [CGM] in children).^15^ In 2023 and 2025, NICE and NHS England also updated the guidance on hybrid closed loop (HCL) pump therapy (HCL) in children and adults.^16^, ^17^ This enabled paediatric diabetes teams to access HCL for patients under 18 years with full re‐imbursement. However, there are still additional costs to families, specifically around mobile phone access for synchronising with HCL, which may affect access and uptake. There has been a significant rise in insulin pump use across all ethnicities, most markedly in Black children (see Table S1). Black children have had a sixfold increase in the proportion using insulin pumps over 10 years (from 5.5% to 33%), while White children saw a 2.5 fold increase in the proportion of insulin pump use (from 20.3% to 49.6%) over a similar period.^11^, ^13^ Children from the most deprived quintile showed an almost threefold increase in the proportion of pump use (from 13.2% to 38.8%) while children from the least deprived quintile showed a 2.3 fold increase in the proportion of pump use (from 21.2% to 50.7%) in the same period.^11^

In 2016, Khanolkar et al. demonstrated from the 2012–2013 National Paediatric Diabetes Audit (NPDA) data set that children and young people of Black ethnicity had the highest HbA1c levels compared to their White peers and that ethnicity was independently associated with glycaemic management even when adjusted for SES and technology (insulin pump) use.^13^

We hypothesised over the intervening 10 years, inequalities in health outcomes would have changed across sociodemographic groups. The primary aim of this study was to compare the HbA1c outcomes between 2012–2013 and 2022–2023 to investigate if there have been any reductions in the HbA1c and pump use inequalities previously demonstrated for lower SES and minority ethnic groups in glycaemic management and technology use.

## 2 METHODS

The study uses a nationwide, population‐based register that is estimated to include >95% of all young people with Type 1 diabetes under 19 years of age in England and Wales with reliable measures of ethnicity and deprivation in England and Wales.

Data were obtained from and analysed in collaboration with the NPDA for England and Wales managed by the Royal College of Paediatrics and Child Health (RCPCH).^18^ The National Paediatric Diabetes Audit (NPDA) is commissioned by the Healthcare Quality Improvement Partnership (HQIP) on behalf of the NHS in England and Wales. The NPDA is a National Clinical Audit Programme that started in 2003 and reached 100% participation of all Paediatric Diabetes Units delivering diabetes care [PDU] by 2012. It includes demographic, HbA1c and pump use data on almost all (>95%) children with Type 1 diabetes and treated at one of the 172 PDUs across England and Wales.^11^ Participation in the NPDA allows units to benchmark their performance. In addition, Best Practice Tariff (BPT) provides an incentivised payment to hospital trusts that deliver care of a specified quality, as evidenced by their participation and performance in the NPDA.^19^, ^20^ This study was based on data collected during the 2022–2023 audit year (01.04.2022–31.03.2023) and compared to data from 2012–2013 audit year.^11^, ^13^

### 2.1 Study population

Inclusion criteria comprised:

- Diagnosis of Type 1 diabetes (patients had to have been diagnosed for at least 6 months to be included to ensure stabilization of glycaemic levels [HbA1c]).
- Age <19 years on first day of audit period.
- Minimum of one visit to a clinic during the audit year with a recorded HbA1c and data recorded for gender, date of diabetes diagnosis and date of birth, and postcode and ethnicity.

### 2.2 Study measurements

#### 2.2.1 Outcomes of interest

The primary outcome of interest was glycaemic levels as measured by HbA1c (mmol/mol). Median HbA1c was calculated from all visits in the audit year for each included young person. HbA1c measurements taken less than 90 days after diagnosis were excluded.

The secondary outcome was pump use versus other treatment options. The primary exposure/independent variables were ethnicity and SES.

##### Pump usage

Insulin treatment was recorded at the time of HbA1c measurement as either pump therapy (continuous subcutaneous insulin infusion, CSII) or daily injections (non‐pump therapy). We did not distinguish between manual or hybrid closed loop CSII as this sub‐analysis was not available in the 2012–2013 data set. We did not analyse CGM use in this work on this basis.

#### 2.2.2 Exposure

The exposure variables for modelling glucose levels were (a) ethnicity, (b) socio‐economic status.

##### 2.2.2.1 Ethnicity

Participants (or their parents) are asked to self‐identify ethnicity when first attending clinic and this is subsequently updated at patient or parent request. As per the Information Standards Board for Health and Social Care, there are 15 ethnicity categories as options as well as the option to decline identifying ethnicity. For this study, these 15 ethnic categories were collapsed into 7 groups (White, Asian, Black, Mixed, Other, “Not known” [where the patient had not been asked or the patient was not in a condition to be asked], and “Not Stated” [those who were asked but declined to provide an ethnic status]). Chinese ethnicity census data has been included in the ‘Other’ category to align with NHS dictionary standards.

##### 2.2.2.2 Socio‐economic status

Socio‐economic status was derived from the English Indices of Multiple Deprivation (IMD) 2019 for England and Welsh Indices of Deprivation 2019 for Wales based on each child or young person's postcode.^21^, ^22^ The postcode is mapped to its Lower Super Output Area (LSOA) and ranked within each country, with rank 1 being most deprived. IMD rank scores within each country were grouped into quintiles for final analysis (first and fifth quintiles corresponding to most and least deprived respectively for England and Wales (general population) and then quintile 1 for England grouped with quintile 1 for Wales and so on). IMD scores are calculated at the level of lower‐layer super output areas, with each area comprising ~1500 individuals on average.^22^

The demographics of everyone in the reference population, including those excluded owing to missing data or answers not given, are provided in Tables S3 and S4.

###### Country

A separate analysis of country was undertaken (England vs. Wales).

###### Co‐variates

The co‐variates analysed were age, gender and duration of diagnosis.

Age of diagnosis was calculated by subtracting date of diagnosis from date of birth. Age was calculated by subtracting the date of birth from the first day of the audit year (01.04.2022).

Duration of diagnosis was calculated by subtracting the date of diagnosis from the first day of the audit year (01.04.2022).

The last recorded entries in the audit year for ethnicity, postcode were used in calculating ethnicity and SES ascertainment. Pump status at last HbA1c measurement was used in the analysis. Gender and ethnicity measures were self‐identified with clinicians.

### 2.3 Statistical analysis

Continuous variables are presented as means with standard deviations (SD) and categorical variables are presented as frequencies. Differences between continuous variables across multiple variables were assessed using a Kruskal–Wallis test, and between binary variables (including the interaction between deprivation and country) using a $\chi^{2}$ test. NPDA use medians per patient and means to compare between groups within the national data set.^18^

Multivariable linear regression models used median HbA1c as the dependent outcome based on the median HbA1c value across all measures for the individual over the year was used. Ethnicity and SES were considered the primary exposure/independent variables for HbA1c. A forward stepwise selection process was used to ascertain the importance of the interaction between ethnicity and SES, age during the audit year (years), sex, age at diagnosis (years) and pump usage (all included as exposure/independent variables). Model fit of the models was compared using R,^2^ which represents the proportion of variation in HbA1c explained by the model, and the inclusion of any one parameter ascertained through likelihood ratio tests. Where multiple independent variables could be added at any one step, the variable which accounted for the most heterogeneity in the data was included. Models used only age in audit and age at diagnosis to ensure no issues with multicollinearity.

An analogous method to that described for HbA1c was undertaken for the analysis of pump usage, with the material difference that logistic regression models were instead used, owing to the distributional form of the dependent variable. Again, ethnicity and SES were considered the primary exposure/independent variables and a forward stepwise selection process was used to ascertain the importance of the interaction between ethnicity and SES, age during the audit year (years), sex and age at diagnosis (years, all included as exposure/independent variables). The methods for variable determination were the same as those described for HbA1c models.

All statistical analyses were conducted using R Versions 4.2.0 and 4.3.3.^23^

#### 2.3.1 Ethical approval

The NPDA has section 251 approval from the Health Research Authority Confidentiality Advisory Group (HRA CAG) to collect confidential patient information without explicit consent (Reference 24/CAG/0146). As such, ethical approval was not required. For this study, all participants were anonymised, making them unidentifiable to the research team.

## 3 RESULTS

### 3.1 Demographics

During the 2022–2023 audit year, 32,049 children and young people with Type 1 diabetes, aged <19 years had data submitted to the NPDA. Of this cohort, a further 1923 were excluded because they were diagnosed within 6 months before the end of the audit year, diagnosis date was missing, or diagnosis date was erroneous—before date of birth. A further 2207 were excluded because they had missing, unknown or unstated postcode, ethnicity, gender, HbA1c or pump use. This left 27,919 (87.1% of eligible sample) for the regression analysis (See Figure S1).

The mean age of the study sample was 12.1 years (standard deviation (SD) 3.9, range 0–18) (Tables 1 and 2). In comparison to the 2012–2013 data set this was significantly younger (mean age of study sample 12.7 years).^13^ The demographics of everyone in the population, included those excluded owing to missing data or answers not given, are provided in Tables S3 and S4. A comparison between this and Tables 1 and 2 reveals no notable difference, thus suggesting the reasons for missingness within the NPDA dataset were random. Age at diagnosis differed by ethnicity. On average, children from Black and White ethnicity groups both had similar mean diagnosis ages (7.4 years), while children from the Asian, Mixed and Other ethnic groups has significantly younger mean diagnosis age (7.1, 7.1 and 6.9 years, respectively, *p* < 0.001 vs. reference group—White Children).

**TABLE 1:** Characteristics of young people with Type 1 diabetes by ethnic group.

	White	Asian	Black	Mixed	Other	Total
Number	22,984	1999	1186	1010	740	27,919
Age at the beginning of the audit year, mean (SD)^a^ ^,^	12.1 (3.9)	11.8 (4.0)	12.2 (3.9)	11.7 (4.0)	11.8 (4.1)	12.1 (3.9)
Age at diagnosis, mean (SD)	7.4 (4.0)	7.1 (3.9)	7.4 (4.1)	7.1 (3.8)	6.9 (4.1)	7.3 (4.0)
Mean Diabetes duration (years) at start of audit year (SD)	4.3 (3.9)	4.2 (3.9)	4.4 (4.0)	4.1 (3.8)	4.4 (4.1)	4.3 (3.9)
Boys, %	53.0	49.2	50.8	51.6	50.7	52.5
HbA1c mmol/mol, mean (SD)	63.1 (15.8)	64.3 (14.8)	69.9 (18.8)	66.3 (18.0)	62.9 (16.0)	63.5 (16.0)
HbA1c %, Mean (SD)	7.9 (3.6)	8.0 (3.5)	8.5 (3.9)	8.2 (3.8)	7.9 (3.6)	8.0 (3.6)
Percentage achieving recommended target for glycaemic level (=<48 mmol/mol), %	12.7	10.1	8.3	9.9	14.5	12.2
Percentage with suboptimal glycaemic level (48‐58 mmol/mol), %	29.2	24.4	19.0	25.6	28.8	28.3
Percentage with poor glycaemic level (>80 mmol/mol), %	11.7	11.9	22.6	16.0	11.4	12.3
Percentage living in the most deprived quintile, %	19.9	38.2	44.8	27.4	35.7	23.0
Percentage using pump therapy, %	49.6	40.6	34.2	44.1	45.1	48.0

*Note*: Values are mean (SD) unless otherwise stated.

^a^ Age at first day of audit year.

**TABLE 2:** Characteristics of young people with Type 1 diabetes by index of multiple deprivation (IMD).

	Least deprived (IMD Quintile 5)	2nd least deprived (IMD Quintile 4)	3rd least deprived (IMD Quintile 3)	3rd most deprived (IMD Quintile 2)	Most deprived (IMD Quintile 1)	Total
Number	5212	5274	5341	5681	6411	27,919
Age at the beginning of the audit year, mean (SD)^a^ ^,^	12.4 (3.8)	12.1 (3.9)	12.1 (3.9)	12.0 (4.0)	11.9 (4.0)	12.1 (3.9)
Age at diagnosis, mean (SD)	7.5 (4.0)	7.3 (4.0)	7.3 (4.0)	7.2 (4.0)	7.3 (4.0)	7.3 (4.0)
Mean diabetes duration (years) at start of audit year (SD)	4.4 (3.9)	4.3 (3.9)	4.3 (3.9)	4.3 (3.9)	4.2 (3.9)	4.3 (3.9)
Boys, %	53.2	52.4	52.5	52.8	52.0	52.5
HbA1c mmol/mol, Mean(SD)	60.0 (13.7)	61.9 (14.8)	63.2 (16.0)	65.1 (16.3)	66.9 (17.7)	63.6 (16.0)
HbA1c % mean (SD)	7.6 (3.4)	7.8 (3.5)	7.9 (3.6)	8.1 (3.6)	8.9 (3.8)	8.0 (3.6)
Percentage achieving recommended target for glycaemic level (=<48 mmol/mol), %	16.0	14.0	12.9	10.4	8.8	12.2
Percentage with suboptimal glycaemic level (48‐58 mmol/mol), %	34.1	30.2	29.4	24.8	24.0	28.3
Percentage with poor glycaemic level (>80 mmol/mol), %	7.6	9.7	11.7	14.4	16.9	12.3
Percentage using pump therapy, %	53.7	52.4	48.6	46.0	41.0	48.0

*Note*: Values are mean (SD) unless otherwise stated.

^a^ Age at first day of audit year.

Similar findings were seen for age at diagnosis for those from the most deprived vs. least deprived quintiles (7.3 vs. 7.5 years, that is, statistically significant difference; *p* < 0.001 with least deprived as reference group; see Table 2). A comparison of IMD vs. Ethnicity for HbA1c is shown in Table 3.

**TABLE 3:** Characteristics of young people with Type 1 diabetes by both index of multiple deprivation and ethnicity (mean mmol/mol; SD) and [%; SD].

	White	Asian	Black	Mixed	Other
Most deprived	66.6 (17.8) [8.2; 3.8] *n* = 4575	66.3 (15.4) [8.2; 3.6] *n* = 764	70.8 (19.2) [8.6; 3.9] *n* = 531	68.5 (19.1) [8.4; 3.9] *n* = 277	64.5 (15.4); [8.1; 3.6] *n* = 264
Second most deprived	64.6 (16.0) [8.1; 3.6] *n* = 4374	64.9 (15.7) [8.1; 3.6] *n* = 511	70.4 (18.8) [8.6; 3.9] *n* = 376	68.3 (18.4) [8.4; 3.8] *n* = 238	63.4 (14) [8.0; 3.4] *n* = 182.0
Third least deprived	63.0 (15.9) [7.9; 3.6] *n* = 4544	62.1 (13.3) [7.8; 3.4] *n* = 333	66.5 (17.0) [8.2; 3.7] *n* = 148	67.2 (18.0) [8.2; 3.8] *n* = 193	61.9 (19.1) [7.8; 3.9] *n* = 123
Second least deprived	61.7 (14.7) [7.8; 3.5] *n* = 4743	62.3 (12.9) [7.9; 3.3] *n* = 221	70.4 (19.5) [8.6; 3.9] *n* = 79	63.0(16.0) [7.9; 3.6] *n* = 152	61.6 (16.6) [7.8; 3.7] n = 79
Least deprived	59.9 (13.6) [7.6; 3.4] *n* = 4748	60.2 (12.1) [7.7; 3.3] *n* = 170	66.3 (18.0) [8.2; 3.8] *n* = 52	61.5 (15.9) [7.8; 3.6] *n* = 150	58.8 (13.9) [7.5; 3.4] *n* = 92

There was no statistically significant difference between diabetes duration between ethnic groups (White vs. any minority ethnic group).

#### 3.1.1 Ethnicity

Compared with children of White ethnicity, children from all other ethnic groups had higher mean HbA1c (White = 63.1 mmol/mol [7.9%], Black = 69.9 mmol/mol [8.5%], Asian = 64.3 mmol/mol [8.0%], Mixed = 66.4 mmol/mol [8.2%], Other = 62.9 mmol/mol [7.9%]). For all ethnic minority groups compared to the reference group (White) this HbA1c difference was significant (*p* < 0.001). 44.8% of black children and 38.2% of Asian children were in the most deprived quintile (Table 1).

In comparison with the White group, all other ethnicities had significantly higher proportions of children in the most deprived SES group (Asian vs. White, *p* < 0.0001, Black vs. White, *p* < 0.0001). In addition, there were significantly more Black children in the most deprived SES group vs. Asian (*p* = 0.0003). These differences in ethnicity HbA1c outcomes are similar to the 2012–2013 data set^13^

#### 3.1.2 Deprivation

A strong association was seen between HbA1c and SES. HbA1c levels increased as deprivation increased (Table 2). A comparison between this and Table 1 reveals no notable difference, thus suggesting the reasons for missingness within the NPDA data set were random. The most deprived quintile (IMD1) had a significantly higher mean HbA1c of 66.9 mmol/mol (8.3%) (SD 17.7) vs. the least deprived quintile mean HbA1c of 60.0 mmol/mol (7.6%) (SD 13.7, *p* < 0.0001). These differences in deprivation are comparable to the 2012–2013 dataset.^13^

An interaction between deprivation and country was undertaken to ensure there were no differences between England and Wales—this was not statistically significant (*p* > 0.995).

#### 3.1.3 Pump use

Significant differences were observed in the proportion of children on insulin pump therapy by ethnicity. Overall, pump usage is more likely in those of White ethnicity (49.6%) and least likely in Black children (34.2%)—see Table 4, (*p* < 0.0001). Similarly, a gradient of pump use is seen according to SES with the probability of using a pump highest in those from the least deprived quintile (IMD Quintile 5) and lowest in those from the most deprived quintile (IMD quintile 1): Least deprived: 54% pump use; Most deprived: 41% pump use, *p* = 0.0001. However, this does not hold true in Black children, where equally low levels of use (31–36%) were seen across deprivation quintiles, *p* = 0.81 (see Table 4).

**TABLE 4:** Pump usage by SES and ethnicity.

Ethnic group	Most deprived (IMD Quintile 1)	Second most deprived (IMD Quintile 2)	Third least deprived (IMD Quintile 3)	Second least deprived (IMD Quintile 4)	Least deprived (IMD Quintile 5)
White	1972/4575 (43.1%)	2093/4374 (47.9%)	2253/4544 (49.6%)	2516/4743 (53.0%)	2568/4748 (54.1%)
Asian	252/764 (33.0%)	211/511 (41.3%)	146/333 (43.8%)	106/221 (48.0%)	96/170 (56.5%)
Black	182/531 (34.3%)	136/376 (36.2%)	46/148 (31.1%)	25/79 (31.6%)	17/52 (32.7%)
Mixed	121/277 (43.7%)	89/238 (37.4%)	91/193 (47.2%)	76/152 (50.0%)	68/150 (45.3%)
Other	103/264 (39.0%)	82/182 (45.1%)	59/123 (48.0%)	41/79 (51.9%)	49/92 (53.3%)
Any	2630/6411 (41.0%)	2611/5681 (46.0%)	2595/5341 (48.6%)	2764/5274 (52.4%)	2798/5212 (53.7%)

*Note*: numerator = numbers on pump, denominator = total in cohort (ethnicity and deprivation quintile, % of cohort on pump given in brackets).

Analysis of pump usage including an interaction between ethnicity and deprivation quintile was not undertaken owing to the low sample size in some of the 2‐way stratum combined with the low information content of a binary outcome would produce unstable and unreliable estimates. However, there was a significant effect of adding ethnicity into the model; therefore, White ethnicity was significantly associated with pump usage (Table S2: Models 1 and 2).

Both ethnicity and SES were independently associated with HbA1c levels (Table S3: Models 3 and 4). Compared with White groups, most ethnic minority groups (Black, Asian and Mixed) had higher mean HbA1c (*p* < 0.001). The largest difference was seen in Black ethnic group (HbA1c 6.8 mmol/mol higher compared to White ethnic group (95% CI 5.7, 7.9)). SES was significantly associated with glycaemic levels comparing the reference group of the least deprived quintile (IMD 5) with the second least deprived (IMD 4: 2.0 mmol/mol, 95% CI 1.4, 2.5) and to the most deprived (IMD 1: 7.0 mmol/L, 95% CI 6.4, 7.6), Model 4, Table S3.

When socio‐economic status and ethnicity are controlled for (Model 5, Table S3), the estimates for ethnic groups were attenuated. HbA1c differences across deprivation quintiles remains very similar to those in Model 4 which does not control for ethnicity, (Model 5, Table S3) suggesting as before that SES explains more of the variation in HbA1c versus ethnicity.^13^

Adjusting for pump usage shows those who do not use a pump have an HbA1c of 7.6 mmol/mol (95% CI: 7.3, 8.0) higher than those who do not use a pump (*p* < 0.001).

**FIGURE 1:**
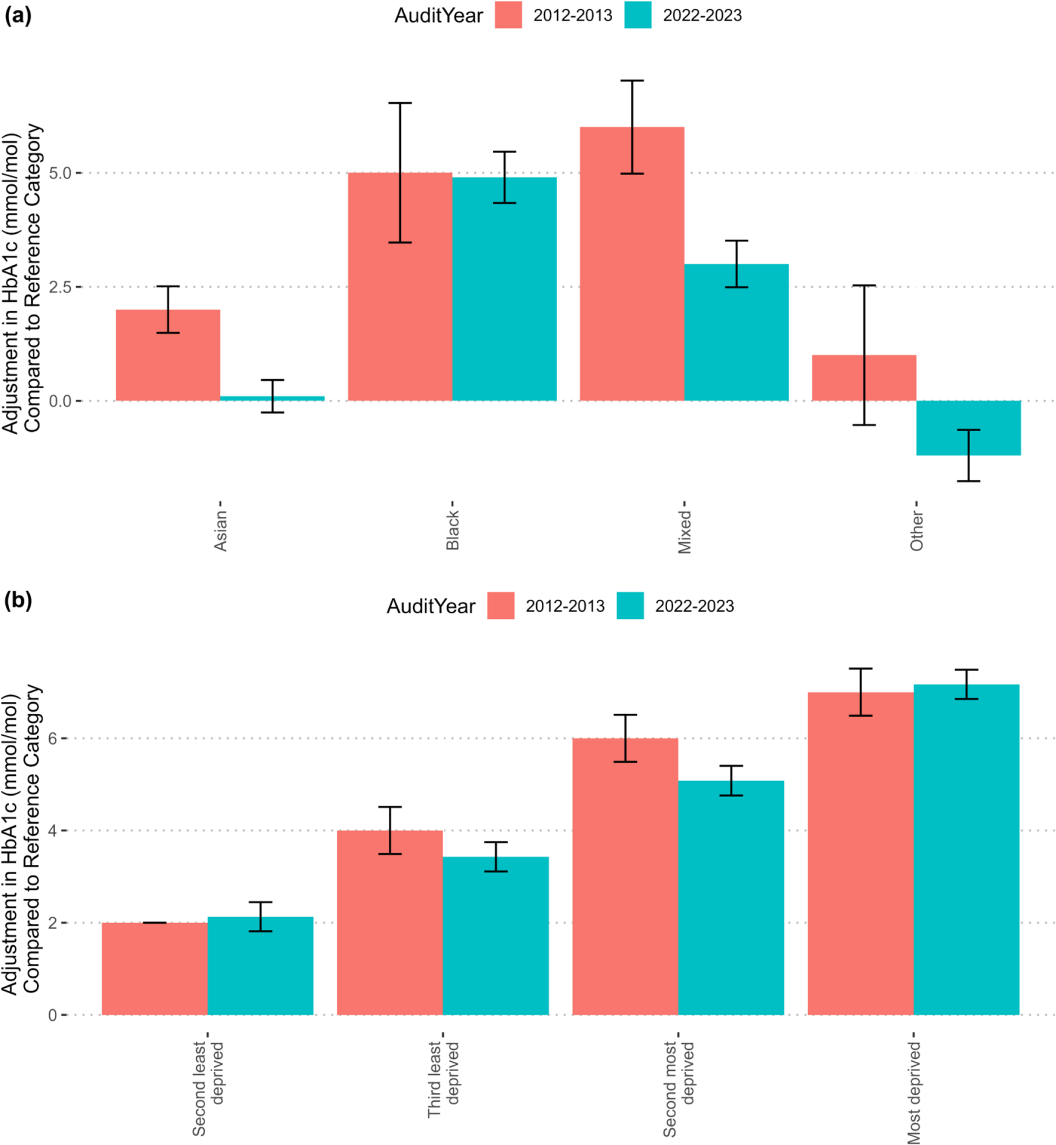
Comparison of HbA1c according to (a) ethnicity (b) deprivation ethnicity in 2012–2013 NPDA dataset vs. 2022–2023 data set. Red bars indicate data from 2012 to 2013, Green bars indicate data from 2022–2023 (main effects from the results given under ethnicity and deprivation fof this manuscript), y axis shows difference in HbA1c compared to reference category (Least deprived or White).

A comparison of the adjustments for the 2022–2023 audit year for ethnicity (White as reference group) compared to the 2012–2013 audit year shows a clinically significant improvement in differences in HbA1c relative to White children for Asian and Mixed children but not for Black children and young people living with Type 1 diabetes (Asian *p* < 0.001, Mixed *p* < 0.001, Black *p* = 0.25), Figure 1a. There were no clinical or statistically significant differences across deprivation quintiles over the 2 audit years (2012/13‐2022/23) (see Figure 1b).

## 4 DISCUSSION

We report inequalities in glycaemic outcomes based on SES and ethnicity from the largest cohort to date of CYP living with Type 1 diabetes in England and Wales.

Our key findings are that ethnicity, SES and insulin pump use remain independently associated with poorer glycaemic management. Black and mixed‐ethnicity children still demonstrate significantly higher HbA1c outcomes compared with their White peers. Lower SES continues to be associated with higher HbA1c levels across all ethnicities. These differences in HbA1c, particularly for Black children, remain clinically significant and show no improvement over the last 10 years. Pump use was associated with improved glycaemic management, as expected.^24^, ^25^ Additionally, despite the significant shift towards pump use across the cohort over the same period, Black children remain less likely to use such technology compared to White children.

Furthermore, for Black children, similar pump take‐up was seen across all deprivation quintiles.

Although the differences seen in terms of age at diagnosis (both between ethnic groups and deprivation quintiles) were statistically significant, they were small and deemed to not be clinically significant (in terms of development stage and school year) across all groups.

Almost a decade ago, Khanolkar et al. described significant and worrying inequalities in both glycaemic management and use of diabetes technology across England and Wales for young people living with Type 1 diabetes with young people across both ethnicity and deprivation.^13^ Reduced uptake of insulin pump uptake or access has also been observed in non‐Hispanic Black young adults in the United States.^26^, ^27^ It has been postulated that this is potentially cultural but doesn't address the issue that equity is not yet being achieved. This is of critical concern to address as it has been shown that pump use improves glycaemic management independent of ethnicity and SES.^13^, ^28^

In a T1D exchange study, there were thought to be additional influences on the HbA1c differences between the White American and African American populations, with only about 50% of the difference explained by higher mean glucose. Other non‐glycaemic factors postulated to impact on higher HbA1c outside of glucose levels included differences in red cell glycation rates, red blood cell lifespan as well as other as yet undefined biological factors.^29^ We did not have real‐time (rt)CGM data to correlate HbA1c in this national cohort but a recent study from the United States showed differences in HbA1c in Black children compared to White children despite adjusting for mean glucose. Equalising technology access seems to diminish HbA1c disparities across SES groups.^30^ The utility of mean blood glucose (MBG) over HbA1c in affecting long‐term outcomes remains to be determined although there are data suggesting in adults at least, MBG is a better predictor than HbA1c for cardiovascular events.^31^ This remains to be studies further in young, ethnically diverse populations.

Our review of the current state of play for CYP living with Type 1 diabetes demonstrates the inequalities in health outcomes that persist despite repeated calls to action to reduce these health inequalities.^12^ It is clear from European and US data that even small reductions in HbA1c can substantially reduce the likelihood of long‐term micro and macro‐vascular complications with a 44% reduction in microvascular complications for every 10% relative reduction in HbA1c in The Diabetes Control and Complications Trial findings and others.^32^, ^33^, ^34^

Despite the challenges, there are ongoing efforts to improve glucose management in underserved communities, including increased use of insulin pump therapy. The introduction of hybrid closed‐loop insulin delivery systems (CSII) for young people with T1D is anticipated to have a significant positive impact on glycaemic outcomes. Going forward there needs to be better understanding on why pump uptake is relatively low in Black children compared to other ethnic minority groups and positive strategies to address this. The most recent NPDA dashboard data for 2025 suggests that across England and Wales there remains ongoing gaps for both HbA1c and pump usage. 49.9% of White children achieve an HbA1c <58 mmol/L vs. 40.5% of non‐White children (not subdivided further) and 54.2% of least deprived achieve this HbA1c target vs. 43% in the most deprived. Additionally, there is a difference in pump use not only between non‐White and White ethnic groups (63.6% vs. 58.4) although this is not subdivided further but also between least deprived and most deprived (75% vs. 67.4%).^35^ A key limitation is the availability of up‐to‐date validated and clean data for analysis. The NICE TA and NHS England guidance on HCL for children was published in December 2023 and implementation started in Spring 2024. This was after the 2022–2023 audit data and it is anticipated that this will have a significant impact on glycaemic management. Future work is planned to assess the 2025–2026 NPDA data when available, to test if the inequality gap has reduced based on availability of HCL. The validated complete year data for 2025–2026 will be available in 2027. Current summary data from the NPDA dashboard indicates a gap remains in uptake on diabetes technology between ethnic groups, however more in‐depth analysis of these data is required to assess the impact.^36^

Ongoing research and policy efforts are needed to bridge these gaps and ensure that all young people with Type 1 diabetes have equity in optimal care. Addressing these inequalities is essential to improving long‐term health outcomes for young people with Type 1 diabetes.

## 5 STRENGTHS AND LIMITATIONS

The major strength of this study is its national representation of Type 1 diabetes in young people across England and Wales.

Data were missing for 4130 patients. This subset was analysed for key demographic characteristics and outcomes, where no differences were seen compared to those where critical data (deprivation and/or ethnicity) were available. We therefore infer that it is a reasonable assumption that they were missing at random. We did not undertake a sub‐analysis by unit to see if there were regional differences based on proportions of ethnic minority populations.

## AUTHOR CONTRIBUTIONS

R.P.D. wrote the manuscript. V.H. led the statistical analysis. R.P.D. and V.H. developed the final statistical models. V.H., C.B., S.P.P. and A.K. undertook the statistical analysis. R.F. supported initial modelling of data and analysis plan development and commented on final manuscript J.T.W., V.H., A.K., S.P.P., R.F. and R.P.D contributed to discussions and reviewed and edited the final manuscript. The corresponding author attests that all listed authors meet authorship criteria and that no others meeting the criteria have been omitted. C.B., S.P.P. and A.K. had full access to all the data in the study. R.P.D. and V.H. are the guarantors of this work and, as such, take responsibility for the integrity of the data and the accuracy of the data analysis.

## FUNDING INFORMATION

No funding has been received for this work. RPD is supported by NIHR Award (Ref NIHR304587) RF is funded by UKRI (ES/W012227/1). The NPDA is commissioned by the Healthcare Quality Improvement Partnership (HQIP) and funded by the NHS in England and Wales.

## CONFLICT OF INTEREST STATEMENT

RPD has received honoraria from Sanofi (participation in advisory board for Teplizumab) and speaker fees from Sanofi and Sandoz. No other conflicts for co‐authors.

## Supporting information


**Figure S1.** CONSORT diagram showing number of participants analysed.

**Table S1.** Summary of HbA1c and Pump Use between 2012–2013 and 2022–2023 NPDA audit years.^13^
**Table S2.** Results from multivariate linear regression –assessing associations between ethnicity, SES and odds of pump usage in children with Type 1 diabetes in England and Wales in 2022–23.
**Table S3.** Characteristics of young people with Type 1 diabetes by Ethnic Group, incorporating those excluded owing to missing data. Table legend values are mean (SD) unless otherwise stated, *Age at first day of audit year.
**Table S4.** Characteristics of young people with Type 1 diabetes by Index of Multiple Deprivation (IMD), incorporating those excluded owing to missing data. Table legend values are mean (SD) unless otherwise stated, *Age at first day of audit year.

## Data Availability

The data that support the findings of this study are available from the NPDA but restrictions apply to the availability of these data, which were used under license for the current study and therefore are not publicly available. Data are however available from the authors upon reasonable request and with permission of NPDA (via HQIP).
