# Supplementary figures and images for: Mind the gap: Ongoing inequalities in glycaemic levels in young people living with Type 1 diabetes across England and Wales
Source: Diabet Med. 2026 Mar 31;43(8):e70283. doi: 10.1111/dme.70283 (PMC13380334; doi:10.1111/dme.70283)

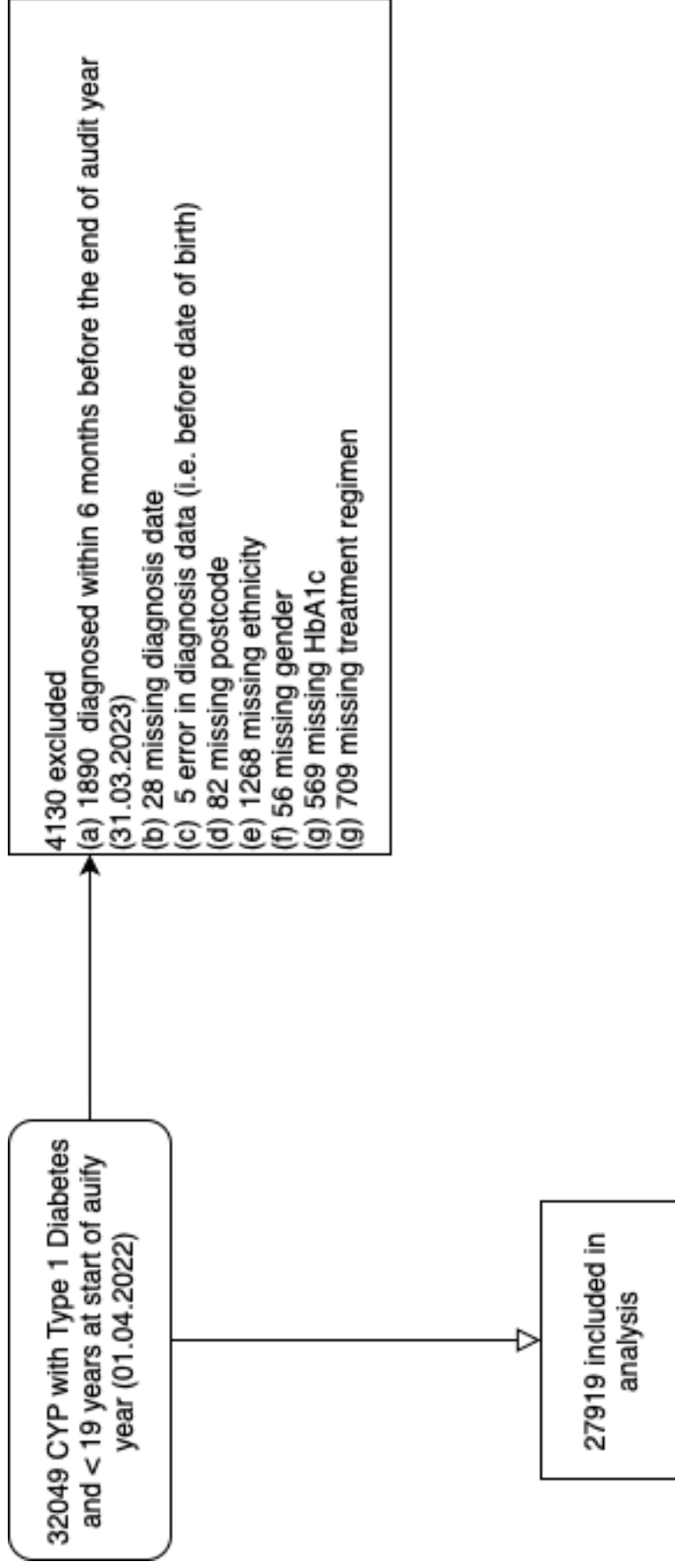

Supplement: Supplementary file 1 — Figure S1. CONSORT diagram showing number of participants analysed. [file DME-43-e70283-s002.pdf]
