# Supplementary material for: Mind the gap: Ongoing inequalities in glycaemic levels in young people living with Type 1 diabetes across England and Wales
Source: Diabet Med. 2026 Mar 31;43(8):e70283. doi: 10.1111/dme.70283 (PMC13380334; doi:10.1111/dme.70283)
Supplement: Supplementary file 2 — Table S1. Summary of HbA1c and Pump Use between 2012–2013 and 2022–2023 NPDA audit years. 13 Table S2. Results from multivariate linear regression –assessing associations between ethnicity, SES and odds of pump usage in children with Type 1 diabetes in England and Wales in 2022–23. Table S3. Characteristics of young people with Type 1 diabetes by Ethnic Group, incorporating those excluded owing to missing data. Table legend values are mean (SD) unless otherwise stated, *Age at first day of audit year. Table S4. Characteristics of young people with Type 1 diabetes by Index of Multiple Deprivation (IMD), incorporating those excluded owing to missing data. Table legend values are mean (SD) unless otherwise stated, *Age at first day of audit year. [file DME-43-e70283-s001.docx]

**Supplementary Tables:**
**Supplementary Table 1: Summary of HbA1c and Pump Use between 2012-2013 and 2022-2023 NPDA audit years** *^12^*

|  | **Mean HbA1c mmol/mol (%)** | | | | **Pump Use (%)** | | | |
| --- | --- | --- | --- | --- | --- | --- | --- | --- |
| **NPDA Audit Year** | **Most deprived Quintile** | **Least Deprived Quintile** | **Black** | **White** | **Most deprived Quintile** | **Least Deprived Quintile** | **Black** | **White** |
| 2012-2013 | 77.0 (9.2) | 70.0 (8.6) | 80.0 (9.5) | 73 (8.8) | 13.2 | 21.2 | 5.5 | 20.3 |
| 2022-2023 | 66.9 (8.3) | 60.0 (7.6) | 69.9 (8.5) | 63.1 (7.9) | 38.8 | 50.7 | 33 | 49.6 |

**Supplementary Table 2: Results from multivariate linear regression –assessing associations between ethnicity, SES and odds of pump usage in children with type 1 diabetes in England and Wales in 2022-23**

|  | **Model 1** Effect of ethnicity only | **Model 2** Effect of deprivation only |
| --- | --- | --- |
| **Ethnicity** |  |  |
| White | *Reference* |  |
| Asian | 0.69 (95% CI: 0.63, 0.76) |  |
| Black | 0.53 (95% CI: 0.47, 0.60) |  |
| Mixed | 0.80 (95% CI: 0.71, 0.91) |  |
| Oher | 0.83 (95% CI: 0.72, 0.97) |  |
| **Deprivation Quintile** |  |  |
| Least Deprived |  | *Reference* |
| Second Least Deprived |  | 0.95 (95% CI: 0.88, 1.0) |
| Third Least Deprived |  | 0.82 (95% CI: 0.76, 0.88) |
| Second Most Deprived |  | 0.73 (95% CI: 0.68, 0.79) |
| Most Deprived |  | 0.60 (95% CI: 0.56, 0.65) |

**Supplementary table 3: Characteristics of young people with Type 1 Diabetes by Ethnic Group, incorporating those excluded owing to missing data**

|  | White | Asian | Black | Mixed | Other | Not stated | Not known | Total |
| --- | --- | --- | --- | --- | --- | --- | --- | --- |
| Number | 23261 | 2022 | 1199 | 1021 | 752 | 737 | 483 | 29475 |
| Age at the beginning of the audit year, Mean (SD) *† | 12.1 (3.9) | 11.8 (4.0) | 12.2 (3.9) | 11.7 (4.0) | 11.8 (4.1) | 11.8 (4.1) | 10.5 (4.1) | 12.0 (3.9) |
| Age at diagnosis, Mean (SD) | 7.4 (4.0) | 7.1 (4.0) | 7.4 (4.1) | 7.2 (3.9) | 7.0 (4.1) | 8.2 (4.1) | 8.3 (4.1) | 7.4 (4.0) |
| Boys, % | 52.9 | 49.1 | 50.8 | 51.5 | 50.5 | 52.4 | 51.3 | 52.4 |
| HbA1c mmol/mol, Mean(SD) | 63.1 (15.8) | 64.3 (14.8) | 69.9 (18.8) | 66.3 (18.0) | 62.9 (16.0) | 62.0 (15.0) | 61.7 (15.6) | 63.5 (16.0) |
| Percentage achieving recommended target for glycaemic level (=<48mmol/mol), % | 12.7 | 10.0 | 8.3 | 9.9 | 14.4 | 15.5 | 16.8 | 12.4 |
| Percentage with suboptimal glycaemic level (48-58mmol/mol), % | 29.1 | 24.4 | 19.0 | 25.5 | 28.9 | 30.4 | 26.3 | 28.2 |
| Percentage with poor glycaemic level (>80mmol/mol), % | 11.7 | 11.8 | 22.6 | 16.1 | 11.7 | 11.5 | 7.9 | 12.2 |
| Percentage living in the most deprived quintile, % | 19.9 | 37.9 | 44.8 | 27.4 | 35.9 | 20.6 | 22.6 | 22.9 |
| Percentage using Pump Therapy, % ^Ψ^ | 49.6 | 40.5 | 34.2 | 44.1 | 45.1 | 37.3 | 33.4 | 47.5 |

***Table Legend*** *Values are mean (SD) unless otherwise stated * Age at first day of audit year*

**Supplementary table 4: Characteristics of young people with Type 1 Diabetes by Index of Multiple Deprivation (IMD), incorporating those excluded owing to missing data**

|  | Least deprived (IMD Quintile 5) | 2^nd^ least deprived (IMD Quintile 4) | 3rd least deprived (IMD Quintile 3) | 3rd most deprived (IMD Quintile 2) | Most deprived (IMD Quintile 1) | Total |
| --- | --- | --- | --- | --- | --- | --- |
| Number | 6749 | 5988 | 5632 | 5568 | 5538 | 29475 |
| Age at the beginning of the audit year, Mean (SD) *† | 11.9 (4.0) | 11.9 (4.0) | 12.0 (3.9) | 12.0 (3.9) | 12.3 (3.9) | 12.0 (3.9) |
| Age at diagnosis, Mean (SD) | 7.3 (4.0) | 7.3 (4.0) | 7.4 (4.0) | 7.3 (4.0) | 7.6 (4.0) | 7.4 (4.0) |
| Boys, % | 52.2 | 52.5 | 52.4 | 52.1 | 52.8 | 52.4 |
| HbA1c mmol/mol, Mean(SD) | 66.9 (17.6) | 65.1 (16.3) | 63.1 (15.9) | 61.9 (14.9) | 60.0 (13.8) | 63.5 (16.0) |
| Percentage achieving recommended target for glycaemic level (=<48mmol/mol), % | 8.9 | 10.6 | 13.2 | 14.1 | 16.1 | 12.4 |
| Percentage with suboptimal glycaemic level (48-58mmol/mol), % | 23.9 | 24.9 | 29.4 | 30.1 | 34.1 | 28.2 |
| Percentage with poor glycaemic level (>80mmol/mol), % | 16.8 | 14.3 | 11.6 | 9.7 | 7.7 | 12.2 |
| Percentage using Pump Therapy, % | 40.6 | 45.5 | 48.1 | 51.8 | 53.2 | 47.5 |

***Table Legend*** *Values are mean (SD) unless otherwise stated, * Age at first day of audit year*
